# Microglia-Derived Extracellular Vesicles Enhance Oligodendrocyte Maturation by Transcriptionally Regulating Mitochondrial Molecular Pathways

**DOI:** 10.1007/s10571-025-01612-7

**Published:** 2025-10-21

**Authors:** Stefano Raffaele, Marta Lombardi, Davide Marangon, Maria P. Abbracchio, Davide Lecca, Claudia Verderio, Marta Fumagalli

**Affiliations:** 1 Department of Pharmaceutical Sciences, Università Degli Studi Di Milano, Via Balzaretti 9, 20133 Milan, Italy; 2 National Research Council of Italy, Institute of Neuroscience (IN-CNR), Via Follereau 3, 20854 Vedano Al Lambro, MB Italy

**Keywords:** Microglia, Oligodendrocytes, OPCs, Extracellular vesicles, Mitochondria, Remyelination, Neuroinflammation

## Abstract

Degeneration of myelinating oligodendrocytes and the resulting breakdown of the myelin sheath are key drivers of neurodegeneration and disability across numerous central nervous system pathologies. Thus, a compelling strategy to preserve neuronal function is to promote endogenous myelin repair by oligodendrocyte precursor cells (OPCs). Extracellular vesicles (EVs) secreted by pro-regenerative microglia have been shown to enhance OPC maturation and remyelination across different experimental models. Yet, the mechanisms by which microglia-derived EVs exert their beneficial effects on OPCs are not fully understood. In this study, we performed transcriptomic profiling of primary murine OPCs treated during differentiation with EVs obtained from donor microglia following stimulation with pro-inflammatory cytokines (i-EVs), interleukin-4 (IL4-EVs), or mesenchymal stem cells in the presence of the inflammatory cocktail (MSC-EVs; GEO accession number: GSE304130). Compared to controls, IL4-EVs and MSC-EVs induced robust changes in gene expression, whereas i-EVs elicited far fewer alterations. Using bioinformatic analyses, we identified the molecular pathways significantly modulated by microglial EVs, revealing a large overlap between IL4-EV and MSC-EV targets. Notably, many of these shared pathways centered on mitochondrial function and bioenergetic metabolism. Upstream regulatory network inference further pinpointed candidate transcription factors and kinases that may drive EV-induced transcriptional reprogramming in OPCs. Hence, our findings indicate that rewiring mitochondria-associated pathways is a core mechanism underlying the pro-differentiation effects of microglial EVs on OPCs. Elucidating these intracellular circuits will open new avenues for developing EV-based or mitochondria-targeted remyelinating therapies.

**Graphical abstract:**

**Figure:**
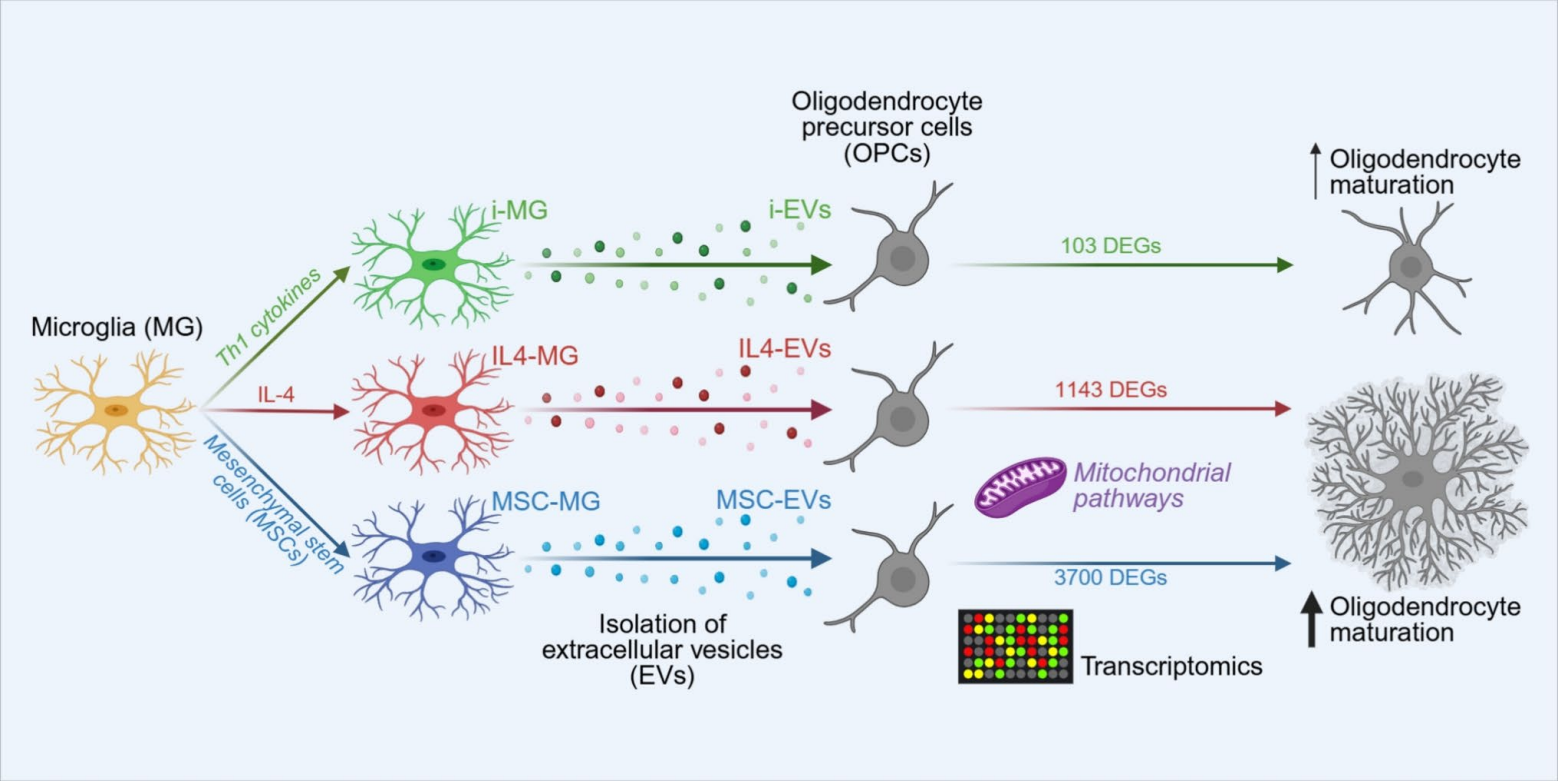
Transcriptional effects of microglia-derived extracellular vesicles on oligodendrocyte maturation. Transcriptomics profiling revealed that extracellular vesicles derived from pro-regenerative microglia (IL4-EVs and MSC-EVs) reprogram oligodendrocyte precursor cells (OPCs) by modulating mitochondrial and bioenergetic pathways, promoting their maturation. In contrast, pro-inflammatory i-EVs induce minimal transcriptional changes and have a limited impact on OPC differentiation

**Supplementary Information:**

The online version contains supplementary material available at 10.1007/s10571-025-01612-7.

## Introduction

Oligodendrocyte dysfunction and myelin damage have recently been recognized as critical contributors to the pathogenesis of various neurodegenerative diseases. Beyond classic demyelinating disorders like multiple sclerosis, emerging evidence implicates disrupted myelin homeostasis in ischemic stroke, Alzheimer’s disease, Parkinson’s disease, and amyotrophic lateral sclerosis, where white matter integrity is compromised even before symptomatic manifestations and accelerates neuronal loss (Raffaele et al. 2021a; Molina-Gonzalez et al. 2022; Cheng et al. 2024; Festa et al. 2024; Kedia and Simons 2025). Furthermore, extensive literature implicates degeneration of oligodendrocyte precursors cells (OPCs) in diffuse white matter lesions of premature babies, and associated cognitive, behavioral, and social defects (Back 2017). These findings underscore that maintaining oligodendrocyte health and myelin integrity is essential not only for classic demyelinating conditions but also for a broad spectrum of neurological pathologies.

OPCs are the principal drivers of myelin maintenance and repair in both the developing and adult central nervous system (CNS). In response to demyelinating injury, OPCs proliferate, migrate into the lesion site, differentiate into mature oligodendrocytes, and ensheathe denuded axons with new myelin sheaths (Franklin and Ffrench-Constant 2017). Successful remyelination hinges on OPCs’ ability to upregulate energy‐sensitive programs, synthesize myelin proteins and lipids, and coordinate extensive membrane assembly (Rosko et al. 2019; Marangon et al. 2020, 2022). However, in chronic demyelinating diseases such as multiple sclerosis and in preterm babies, OPC differentiation often stalls, despite abundant OPC presence, leading to incomplete or failed remyelination and subsequent axonal degeneration (Molina-Gonzalez et al. 2022). Unraveling the extrinsic and intrinsic cues that govern OPC fate is therefore crucial for developing therapies aimed at boosting remyelination and preserving neuronal integrity.

Microglia, the resident immune cells of the central nervous system, play a pivotal role in shaping the remyelination environment (Raffaele and Fumagalli 2022; Kent and Miron 2023). Under homeostatic conditions, microglia support myelin debris clearance, secrete trophic factors, and maintain myelin integrity. However, upon injury or chronic inflammation, microglia can adopt diverse reactive states that either facilitate or hinder OPC maturation (Lloyd and Miron 2019). A central role in this process is played by the microglial secretome, composed by cytokines, reactive oxygen species, anti‐inflammatory mediators, growth factors, and enzymes that can either trigger oligodendrocyte disruption or promote OPC survival and myelin repair. Thus, the balance between heterogeneous microglial states critically influences whether OPCs can progress toward fully myelinating oligodendrocytes in lesioned tissue.

A peculiar mechanism by which microglia communicate with OPCs and regulate their differentiation is the release of extracellular vesicles (EVs), membrane-bound nanoparticles loaded with lipids, proteins, and nucleic acids whose composition accurately reflects the reactive state of donor cells (Gualerzi et al. 2021; Gabrielli et al. 2022b). Microglial reactive state can be grossly altered by different stimuli in vitro as a tool to study changes in the molecular composition and biological effects of the EVs they release*.* Specifically, we showed that exposure to Th1 cytokines (IL-1β, TNF, and IFNγ) drives a pro-inflammatory microglial state (i-MG), whereas interleukin-4 (IL4) biases microglia toward a more pro-regenerative phenotype (IL4-MG). Moreover, co-culture of microglia with mesenchymal stem cells (MSCs) in the presence of Th1 cytokines further re-establishes regenerative traits while maintaining an inflammatory signature (MSC-MG) (Lombardi et al. 2019). When administered in vivo, EVs isolated from IL4-MG and MSC-MG, but not i-MG, were found to promote OPC maturation and myelin repair in white matter lesions induced by either lysolecithin injection or experimental ischemic stroke (Lombardi et al. 2019; Raffaele et al. 2021b). These results were further confirmed by in vitro experiments, showing that microglia-derived EVs directly enhance the differentiation of primary OPCs and their ability to myelinate neuronal tracts in co-culture (Lombardi et al. 2019, 2024; Raffaele et al. 2021b). Several components of the microglial EV cargo have been shown to contribute to their pro-differentiating effects on recipient OPCs, including cytokines like TNF (Raffaele et al. 2020, 2021b), lipids like endocannabinoids (Lombardi et al. 2019, 2024) and cholesterol (Vanherle et al. 2023), and nucleic acids, such as miRNAs (Li et al. 2022). Nevertheless, despite established effects of microglial EVs on OPC differentiation, the precise transcriptional programs and molecular pathways triggered by distinct microglial EV subsets in target OPCs remain poorly defined. Understanding these responses is crucial, as it may inform the development of EV-based strategies to promote OPC differentiation and remyelination in demyelinating conditions. To fill this gap, here we performed a comprehensive transcriptomic profiling of primary murine OPCs exposed to EVs from pro-inflammatory (i-EVs) or pro-regenerative (IL4-EVs and MSC-EVs) microglia. Moreover, bioinformatic tools have been exploited to investigate the cellular components and molecular pathways significantly modulated by microglial EVs, as well as to predict the upstream transcription factors and kinases involved. In summary, our study revealed that pro-regenerative EVs converge on mitochondrial molecular pathways to drive OPC differentiation, laying a foundation for designing EV-based or mitochondria-targeted strategies aimed at enhancing remyelination in CNS disorders featuring myelin loss.

## Materials and Methods

### Animal Care

All procedures involving animals were conducted in compliance with national (D.L. n.26, 2014) and European (Directive 2010/63/EU) legislation, following ethical approval by the Italian Ministry of Health and the University of Milan Animal Welfare Committee (approval number 868/2016-PR). Experiments adhered to ARRIVE guidelines.

### Primary Microglia Cultures

Primary microglia were isolated from mixed glial cell cultures derived from the brain tissue of C57BL/6 mouse pups at postnatal day 2 (Charles River, Lecco, Italy) and the mixed cultures were maintained for 10 days in medium supplemented with South American fetal bovine serum (Life Technologies, Monza, Italy) that promotes microglia proliferation. Then, microglia were collected by agitation on an orbital shaker (1300 rpm, 40 min) and subsequently re-plated onto poly-l-ornithine-coated tissue culture plates (50 μg/ml, Sigma-Aldrich, Taufkirchen, Germany). Pure microglia cultures (> 98%) were expanded for 48 h and placed in 1% serum medium for 24 h to reduce basal activation. Microglia where then stimulated for 48 h with either a pro-inflammatory cytokine mix, including 20-ng/ml IL-1β (Peprotech, Milan, Italy), 20-ng/ml TNF (Peprotech, Milan, Italy), and 25-ng/ml IFN-ɣ (Sigma-Aldrich, Taufkirchen, Germany), or 20-ng/ml IL4 (R&D, Milan, Italy) to induce pro-inflammatory (i-MG) or pro-regenerative (IL4 MG) phenotypes, respectively (Prada et al. 2018). An additional condition involved microglia co-culture with bone marrow-derived MSCs, obtained from 6- to 8-week-old C57BL/6J mice as described (Zappia et al. 2005; Lombardi et al. 2019, 2024), in a Transwell system (microglia-to-MSCs ratio of 1:1) for 48 h in the presence of pro-inflammatory cytokines as above. All conditions were briefly exposed to ATP to enhance EV secretion. Cell supernatants were collected for EV isolation, whereas donor microglia were harvested for real-time qPCR analysis of phenotypic markers. Time-matched unstimulated microglia cultured in standard medium served as control for qPCR analysis.

### Extracellular Vesicles (EVs) Preparation

Before EV isolation, Transwell supports carrying MSCs and culture medium containing inflammatory cytokines or IL4 were removed, and stimulated microglia were treated with 1-mM ATP (Sigma-Aldrich, Taufkirchen, Germany) for 30 min in KRH buffer (125-mM NaCl, 5-mM KCl, 1.2-mM MgSO4, 1.2-mM KH2PO, 2-mM CaCl2, 6-mM D-glucose, and 25-mM HEPES/NaOH, pH 7.4) to foster EV release (Bianco et al. 2005). Supernatants were centrifuged at 300 × g for 10 min to eliminate detached cells and debris, followed by ultracentrifugation at 100,000 × g for 1 h to collect total EVs, including small and large populations (Gabrielli et al. 2015). All EV quality controls, including analysis of size distribution and concentration by Tunable-Resistive Pulse Sensing, purity by western blotting analysis, and ultrastructure by cryo-EM, are standardized according to the ISEV guidelines (Welsh et al. 2024) and are reported in our previous work (Lombardi et al. 2019; Raffaele et al. 2021b; Gabrielli et al. 2022a). EV pellets were resuspended in OPC culture medium and used immediately after isolation, whereas medium alone without EVs served as control.

### Primary OPC cultures

Primary OPCs were isolated from total brain tissue of P2 C57BL/6 pups (Charles River, Lecco, Italy) using magnetic activated cell sorting (MACS) as described previously (Bonfanti et al. 2020; Raffaele et al. 2021b). Brain tissue was enzymatically dissociated into single-cell suspensions using Papain-based Neural Tissue Dissociation Kit (Miltenyi Biotec, Bologna, Italy). OPCs were sorted via anti-CD140a (PDGFRα) microbeads (Miltenyi Biotec, Bologna, Italy) and plated onto poly-DL-ornithine coated plates in OPC proliferation medium composed of Neurobasal (Life Technologies, Monza, Italy), 2% B27 (Life Technologies, Monza, Italy), 1% L-glutamine (Euroclone, Pero, Italy), 1% penicillin/streptomycin (Euroclone, Pero, Italy), 10 ng/ml PDGF-AA (Sigma-Aldrich, Taufkirchen, Germany), and 10-ng/ml FGF2 (Space Import Export, Milan, Italy). After two days, cells were switched to differentiation medium containing DMEM (Euroclone, Pero, Italy), 1% N-2 supplement (Life Technologies, Monza, Italy), 2% B27, 0.01% BSA (Sigma-Aldrich, Taufkirchen, Germany), 1% L-glutamine, 1% penicillin/streptomycin, and 10 ng/ml T3 (Sigma-Aldrich, Taufkirchen, Germany). After ~ 4 h, cultures were treated with either medium alone (CTRL), i-EVs, IL4-EVs, or MSC-EVs (50 µl/well; recipient OPCs:donor microglia ratio 1:2). Cells were plated in 6-well plates at a density of 300.000 cells/well and collected after 24 h of differentiation (n = 3 independent replicates/group). Cultures consistently showed < 1% astrocyte or microglial contamination.

### RNA Isolation

Donor microglia and recipient OPCs were lysed using TRIZOL® reagent (Life Technologies, Monza, Italy). For microglia samples, total RNA was extracted by Direct-zol™ RNA Micro-Prep (Zymo Research, Irvine, CA, USA) according to the manufacturer’s instructions. For OPC samples undergoing transcriptomics, total RNA was extracted by means of RNeasy Micro kit (Qiagen, Hilden, Germany) following the manufacturer’s protocol.

### RT-qPCR

RNA samples were treated with RQ1 DNase (Promega, Milan, Italy) and 400-ng RNA/sample were reverse-transcribed using the SensiFAST™ cDNA synthesis kit (Bioline, London, UK). TaqMan® Gene Expression Assay reactions were set up with 20-ng cDNA, 250-nM probe (*Arg1* Mm00475988_m1; *Chil3* Mm00657889_mH; *Cspg4* Mm00507257_m1; *Gpr17* Mm02619401_s1; *Il1b* Mm00434228_m1; *Mbp* Mm01266402_m1; *Mrc1* Mm01329359_m1; *Ptgs2* Mm00478374_m1; *Tnf* Mm00443258_m1; Nos2 Mm00440502_m1; *Rpl13a* Mm05910660_g1; Life Technologies, Monza, Italy), and Master Mix 2 × (Life Technologies, Monza, Italy). Reactions were run on a CFX96 Real-Time PCR System (Bio-Rad Laboratories, Segrate, Italy). *Rpl13a* has been used as housekeeping gene for internal normalization. Data were analyzed using the ΔΔCt method and are presented as mean of log2(fold change) ± SE. One-way ANOVA followed by Tukey’s post hoc test was conducted using Prism 10 software (GraphPad, San Diego, CA, USA). Statistical significance was defined as p < 0.05.

### Transcriptomics

Before transcriptomics analysis, RNA concentration and purity were assessed by spectrophotometer (Nanodrop): 260/280 and 260/230 ratios were evaluated. Total RNA integrity was assessed by Agilent Bioanalyzer and the RNA Integrity Number (RIN) was calculated. The RIN ranges from 1 (totally degraded RNA) to 10 (completely intact RNA). The quality of each sample was assured by a RIN ≥ 6 and visual confirmation of clear, distinct 28S and 18S rRNA peaks.

100 ng of RNA was used for the preparation of targets for Clariom™ D mouse arrays according to the GeneChip™ WT Plus Reagent Kit manual. The Clariom™ D mouse arrays (which contain > 66,100 genes, > 214,900 transcripts, > 498,500 exons, > 282,500 exon–exon splice junctions, with > 4,895,600 total probes) were purchased from Thermo Fischer Scientific (Massachusetts, USA). The staining, washing and scanning of the arrays were conducted using a Fluidics 450 station, Command Console Software and GeneChip® Scanner 3000 7G, generating .CEL files for each array (Thermo Fischer Scientific, USA).

The images were scanned by Thermo Fisher GeneChip Command Console (AGCC) and analyzed with the Thermo Fisher GeneChip Expression Console. The quality control of the scanned data was first estimated by confirming the order of the signal intensities of the Poly-A and Hybridization controls using Expression Console Software (Thermo Fischer Scientific, USA).

Raw expression values were imported as Thermo Fisher .CEL files into TAC 4.0 software (Thermo Fisher Scientific, USA). Raw expression values from the Thermo Fisher miRNA 4.0 arrays were analyzed and normalized using the TAC 4.0 software, which includes the Preprocessing, Differentially Expressed Genes (DEGs) Finding and Clustering modules.

For this experiment, a total of 12 .CEL files (3 CTRL, 3 i-EVs, 3 IL4-EVs, and 3 MSC-EVs) were uploaded and normalized in PM (perfect match)-only conditions as a PM intensity adjustment. A Robust Multichip Analysis (RMA) quantification method (Irizarry et al. 2003) was used as a probe set summarization algorithm for log transformation with base 2 (log2) and the Quantile normalization method was chosen to evaluate the preliminary data quality in the Preprocessing module, which functions as a data quality control through the Thermo Fisher Expression Console Software. The mean signal intensities of all genes were obtained using 3 chips from each group.

Hierarchical clustering dendrogram and heatmap were used to visualize global gene expression changes between groups. For each gene, a one-way ANOVA was performed across the groups (CTRL, i-EVs, IL4-EVs, MSC-EVs), and the resulting p values were adjusted for multiple testing using the Benjamini–Hochberg false discovery rate (FDR) method. The 1000 genes with the lowest adjusted p values were selected as top genes and used for heatmap generation. Visualization was performed using the R pheatmap package, applying row-wise scaling to allow comparison of expression differences across samples. Hierarchical clustering was performed on both genes and samples using Euclidean distance and the complete linkage method.

After normalization, the differentially expressed genes (DEGs) in EV-treated samples vs CTRL, satisfying the conditions of the fold change cutoff 1,5 and a FDR corrected p-value < 0,05 from all of the genes probed in the array, were selected (i-EVs: 103 DEGs, IL4-EVs: 1143 DEGs, MSC-EVs: 3700 DEGs) and are included in Supplementary Table 1.

### Gene Set Enrichment Analysis

The lists of DEGs induced by IL4-EVs and MSC-EVs were subjected to functional enrichment analysis using the web-based Metascape toolkit (Zhou et al. 2019), allowing direct comparison of the different datasets through the Multiple Gene Lists tool with default settings. This analysis considered a total of 859 and 1889 DEGs starting from the original 1143 and 3700 DEGs, respectively, after exclusion of all non-coding genes by the software.

Circos plots were generated to show overlapping DEGs across the two datasets and genes sharing the same ontology terms.

The Gene Ontology (GO) Cellular Components and GO Biological Processes databases (Ashburner et al. 2000) were considered to define the enriched subcellular components and biological processes shared by the two datasets, whereas the Reactome Pathway Knowledgebase (Milacic et al. 2024) and Kyoto Encyclopedia of Genes and Genomes (KEGG) (Kanehisa et al. 2023) databases were considered to investigate common enriched molecular pathways. Results are included in Supplementary Table 2.

Protein–protein interaction (PPI) enrichment analysis has been carried out using only physical interactions in STRING (Szklarczyk et al. 2023) and BioGrid (Oughtred et al. 2019) databases. The resultant network contains the subset of proteins that form physical interactions with at least one other member in the list. The Molecular Complex Detection (MCODE) algorithm (Bader and Hogue 2003) has been applied to identify densely connected network components. Enrichment analysis has been applied to each MCODE component independently, and the best-scoring terms by p value have been retained as the functional description of the corresponding components.

### Upstream Regulatory Network Analysis

Upstream regulatory networks were inferred from DEGs using the X2K web tool (Clarke et al. 2018), which integrates transcription factor enrichment analysis, PPI network expansion, and kinase enrichment analysis to identify transcription factors and kinases potentially regulating DEGs induced by microglial EVs in target OPCs. Initially, query gene sets were compared against ChEA3 transcription factor target libraries (Keenan et al. 2019). The resulting protein interactors were then analyzed using the KEA3 database (Kuleshov et al. 2021) to predict kinase–substrate relationships, kinase–protein interactions, and additional associations based on co-expression and co-occurrence data, enabling the identification of upstream regulators potentially driving the observed transcriptional changes.

## Results

### EVs from Distinct Microglial States Differentially Promote OPC Maturation

**Fig. 1:**
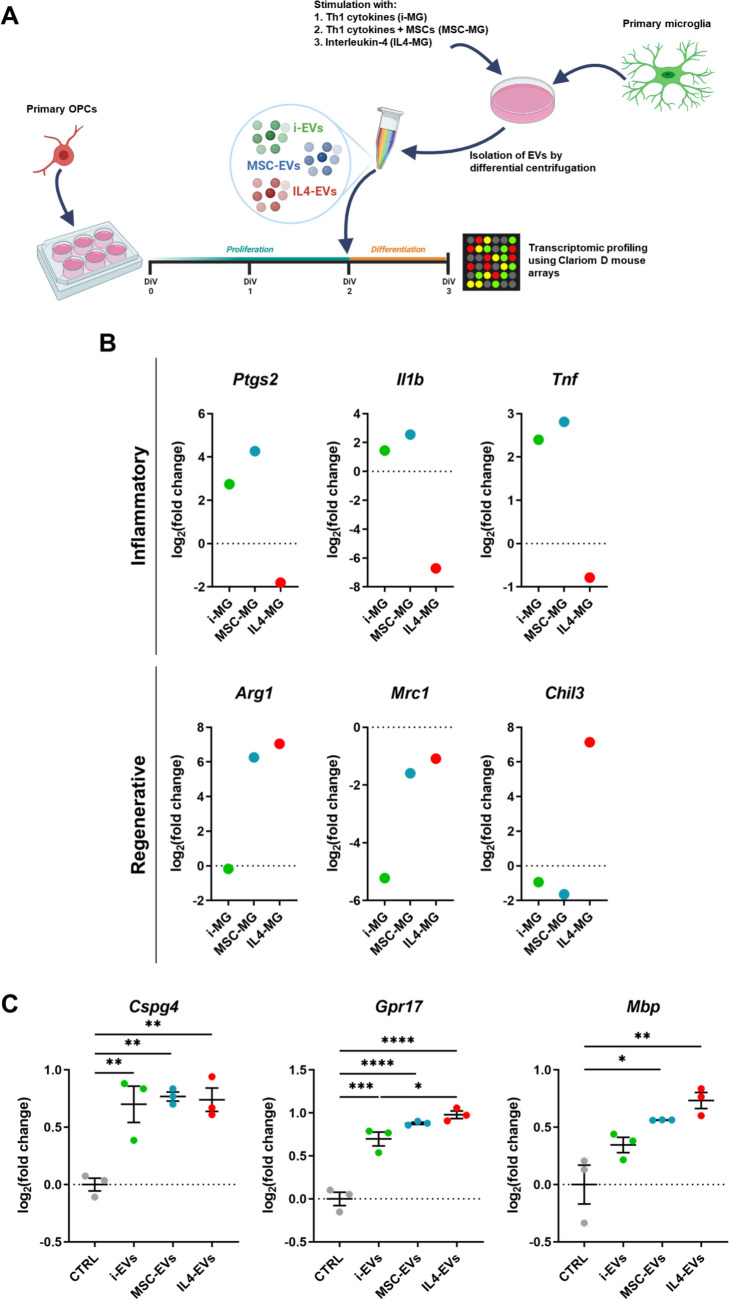
Effects of microglia-derived EVs on OPC maturation. (**A**) Schematic representation of the experimental protocol utilized to evaluate the transcriptomic effects of EVs derived from microglia with different reactive states on recipient OPCs. Created with BioRender.com. (**B**) Relative expression of inflammatory (*Ptgs2*, *Il1b*, *Tnf*) and regenerative (*Arg1*, *Mrc1*, *Chil3*) genes in primary microglia exposed to different stimuli and utilized to collect EVs. Gene expression shown is relative to time-matched unstimulated microglia, indicated by the dashed line. One representative sample per each condition has been analyzed. (**C**) Relative expression of genes related to different oligodendrocyte differentiation stages (*Cspg4* for early OPCs; *Gpr17* for differentiation-committed OPCs; *Mbp* for mature oligodendrocytes) in primary OPC cultures exposed to EVs derived from microglia with different reactive states (*n* = 3). * *p* < 0.05, ** *p* < 0.01, *** *p* < 0.001, **** *p* < 0.0001; One-way ANOVA followed by Tukey’s post-hoc test. MSC: Mesenchymal stem cells; DIV: Days in vitro; EVs: Extracellular vesicles; OPCs: Oligodendrocyte precursor cells; MG: Microglia; IL4: Interleukin 4

Primary microglia have been exposed in vitro to different stimuli to mimic distinct reactive states according to our previous studies (Fig. 1A; (Lombardi et al. 2019)). Briefly, Th1 cytokines were used to induce a pro-inflammatory phenotype (i-MG), while interleukin-4 (IL4-MG) and co-culture with mesenchymal stem cells in the presence of Th1 cytokines (MSC-MG) were utilized to obtain a pro-regenerative phenotype. Notably, qPCR analysis of selected phenotypic markers confirmed the acquisition of the desired reactive state by microglia in these experimental conditions compared to time-matched unstimulated cells (Fig. 1B), in line with our previous studies (Lombardi et al. 2019; Raffaele et al. 2021b). Then, primary murine OPCs were exposed to the EVs obtained from microglia with different reactive states (i-EVs, IL4-EVs, or MSC-EVs) or medium alone (CTRL) for 24 h during differentiation (Fig. 1A). Of note, qPCR analysis confirmed that, compared to CTRL condition, all types of EVs elevated the expression of the early OPC marker *Cspg4* and of the differentiation-committed OPC marker *Gpr17* in recipient differentiating cells. Moreover, at this time point, only IL4-EVs and MSC-EVs, but not i-EVs, significantly increased the expression of the mature myelinating marker *Mbp* (Fig. 1C). These results confirm that a significant increase of OPC differentiation was induced by microglial EVs vs CTRL, with a more pronounced effect of IL4-EVs and MSC-EVs compared to i-EVs, setting the stage for investigating the transcriptional changes underpinning these effects.

### Pro-Regenerative Microglia-Derived EVs Induce Prominent Transcriptional Changes in Recipient OPCs

**Fig. 2:**
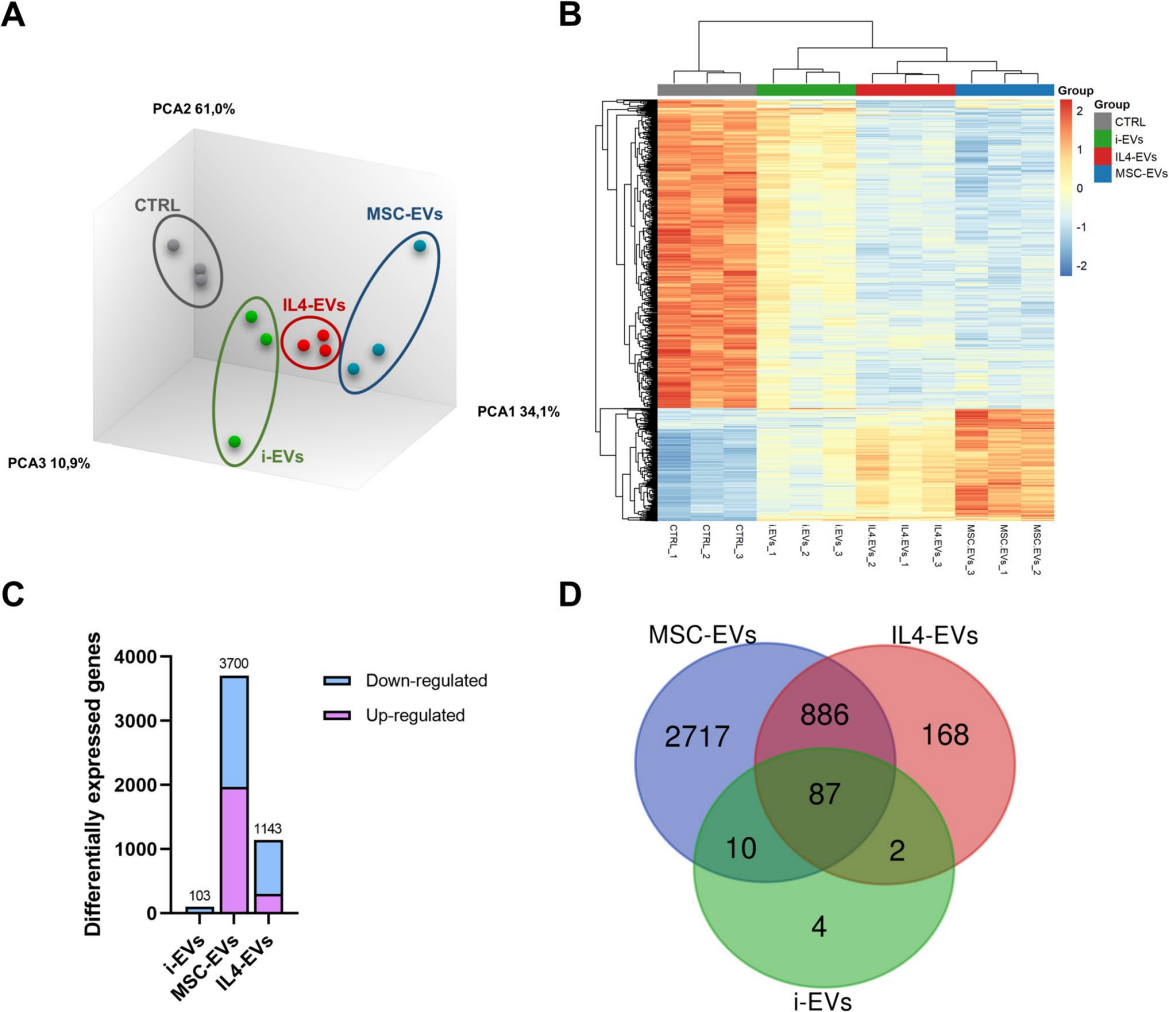
Transcriptomic changes induced by microglia-derived EVs on recipient OPCs. (**A**) Plot showing the results of the principal components analysis. (**B**) Hierarchical clustering dendrogram and heatmap showing global gene expression changes within groups, rows show the z-score of the top 1000 genes. (**C**) Graph showing the number of total, up-regulated, and down-regulated differentially expressed genes (DEGs) in OPCs exposed to i-EVs, MSC-EVs, or IL4-EVs compared to CTRL. (**D**) Venn diagram showing the overlap between the DEGs induced by i-EVs, MSC-EVs, or IL4-EVs compared to CTRL. Numbers refer to the DEGs in each intersection

Next, we evaluated the transcriptomic profiles of OPCs exposed to i-EVs, IL4-EVs, MSC-EVs, or CTRL using Clariom D mouse arrays, containing 65.956 mouse genes. First, to assess the quality of replicates, we performed a principal component analysis (PCA), whose results indicated that the 3 replicates for each condition were concordant (Fig. 2A). Moreover, we checked the global gene expression changes between the samples by means of hierarchical clustering and heatmap analysis, which showed that CTRL samples clustered separately from the others, in line with the presence of transcriptomic changes induced by all types of microglial EVs compared to CTRL (Fig. 2B). Interestingly, we found that samples exposed to IL4-EVs and MSC-EVs grouped within a separate branch from those receiving i-EVs (Fig. 2B), suggesting that protective types of microglial EVs share substantial transcriptional changes compared to CTRL. Conversely, at least within the short timespan evaluated, i-EVs exhibited an intermediate transcriptional profile, showing some alterations compared to CTRL but less pronounced than those induced by protective EVs (Fig. 2B).

Next, to evaluate more in detail the transcriptomic effects induced by each type of microglial EVs compared to CTRL, the differentially expressed genes (DEGs) satisfying the conditions of the fold change cutoff 1.5 and a false discovery rate (FDR) corrected p value < 0.05 were selected from all the genes probed in the array. Results showed that MSC-EVs and IL4-EVs induced substantial transcriptional changes compared to CTRL (3700 and 1143 DEGs, respectively), while i-EVs produced less significant changes (103 DEGs; Fig. 2C, full lists of DEGs in Supplementary Table 1). Representation of the three lists of DEGs by a Venn diagram highlighted that almost all DEGs modulated by i-EVs are shared by the other EV types, and that IL4-EVs and MSC-EVs share a large subset of DEGs, while the majority of genes are unique to MSC-EVs (Fig. 2D). Hence, these results uncovered pronounced transcriptional changes induced by IL4-EVs and MSC-EVs in recipient cells, whereas the effects of i-EVs appeared to be very limited. Based on these considerations, we decided to exclude i-EVs from subsequent analysis and to focus on the comparison between IL4-EVs and MSC-EVs.

### Pro-Regenerative Microglia-Derived EVs Modulate Mitochondria-Related Genes in OPCs

**Fig. 3:**
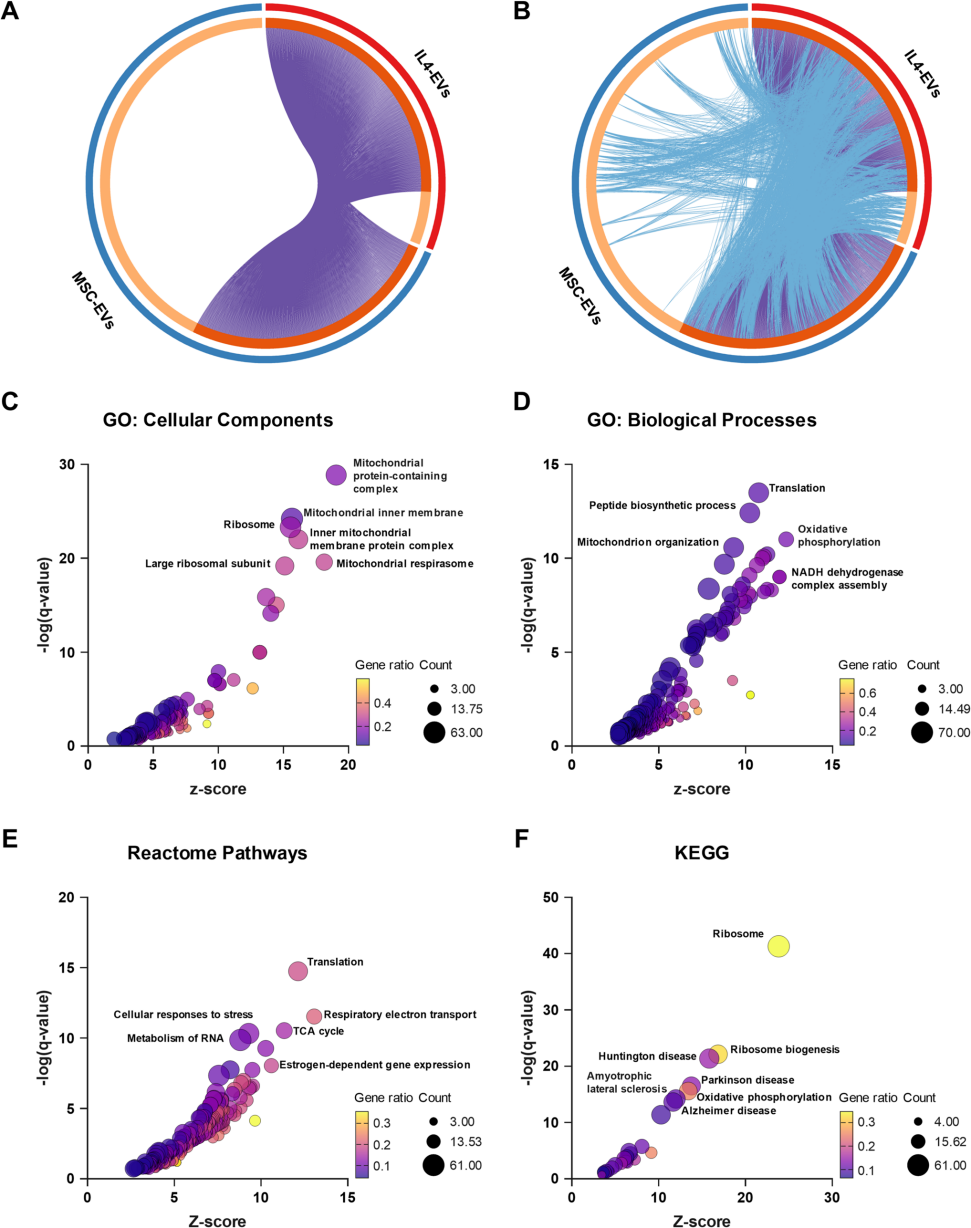
Pathways modulated by microglia-derived EVs in recipient OPCs. (**A-B**) Circos plots showing common DEGs (A) and DEGs sharing the same molecular pathways (B) in OPCs exposed to IL4-EVs or MSC-EVs compared to CTRL. On the outside, each arc represents the identity of each gene list. On the inside, each arc represents a gene list, where each gene member of that list is assigned a spot on the arc. Dark orange color represents the genes that are shared by multiple lists and light orange color represents genes that are unique to that gene list. Purple lines link the same genes that are shared by multiple gene lists. Light blue lines link genes that, although different, fall under the same ontology term. (**C-F**) Bubble plots showing significantly enriched GO: Cellular Components (C), GO: Biological Processes (D), Reactome Pathways (E), and KEGG Pathways (F) shared by OPCs exposed to IL4-EVs or MSC-EVs compared to CTRL. Color code indicates the proportion of genes in each pathway that are enriched in the DEG list (Gene Ratio), whereas bubble size indicates the absolute number of overlapping genes between a given pathway and the DEG list (Count)

To ensure biologically meaningful interpretation, DEG lists were filtered prior to subsequent analysis to exclude non-coding transcripts, including lncRNAs and pseudogenes, retaining 859 and 1889 protein-coding DEGs for IL4-EVs and MSC-EVs, respectively. Circos plots were used to more clearly visualize the overlap in protein-coding DEGs between OPCs treated with IL4-EVs or MSC-EVs compared to the CTRL group (Fig. 3A), as well as to highlight treatment-specific DEGs that converge on shared molecular pathways (Fig. 3B). This approach revealed a strong functional intersection between the two DEG profiles, which not only shared numerous common genes but also a substantial proportion of overlapping molecular pathways (Fig. 3A, B). To get more insight into the functional effects of IL4-EVs or MSC-EVs on recipient OPCs, the datasets of DEGs were then subjected to functional enrichment analysis using the Metascape resource (Zhou et al. 2019). This approach revealed a total of 69 gene ontology (GO): Cellular Components (Fig. 3C), 114 GO: Biological Processes (Fig. 3D), 175 Reactome Pathways (Fig. 3E), and 22 KEGG (Fig. 3F) significantly enriched annotations shared between the two lists of DEGs, confirming a substantial functional overlap between the two conditions. A full list of results obtained from functional enrichment analysis is provided in Supplementary Table 2.

Interestingly, analysis of GO: Cellular Components unveiled the mitochondrial inner membrane and ribosomes as the top compartments enriched in both datasets (Fig. 3C). Accordingly, the top GO: Biological Processes are related to mitochondrial ATP synthesis via oxidative phosphorylation, mitochondrion organization, and protein translation (Fig. 3D). These findings were further corroborated by the enriched Reactome Pathways terms, which were mostly related to mitochondrial respiratory electron transport, mitochondrial metabolism, and protein translation (Fig. 3E). A final validation was provided by interrogating the top enriched KEGG terms, which were related to oxidative phosphorylation and ribosomes, as well as to many neurodegenerative diseases (Huntington, Parkinson, and Alzheimer diseases, amyotrophic lateral sclerosis) characterized by impaired oligodendrocyte differentiation as well as mitochondrial dysfunction (Fig. 3F).

**Fig. 4:**
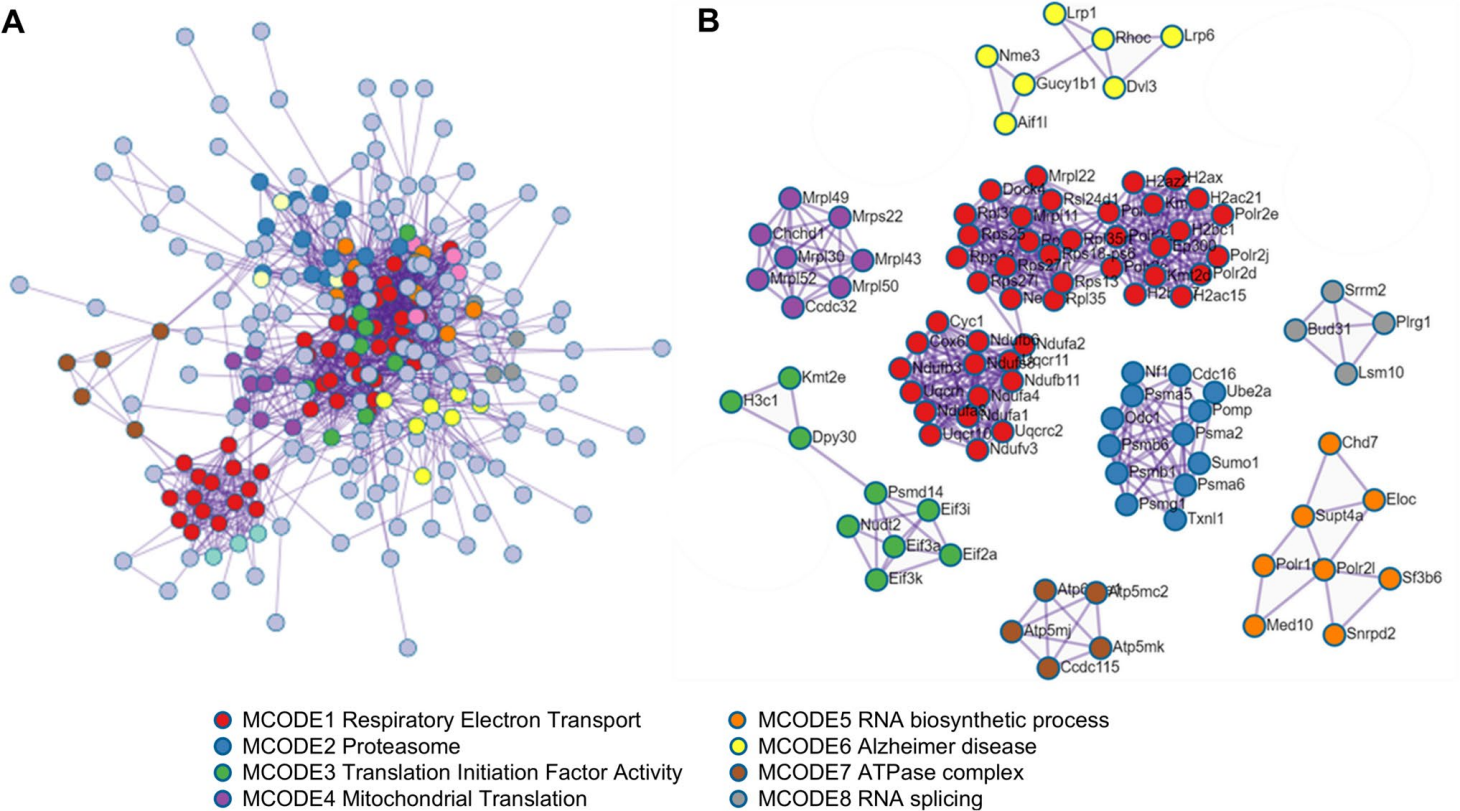
Protein–protein interaction network modulated by microglial EVs in recipient OPCs. (**A-B**) Protein–protein interaction network (A) and MCODE clusters (B) shared by the DEG lists of OPCs exposed to IL4-EVs or MSC-EVs compared to CTRL

Next, Metascape was used to analyze the protein–protein interaction (PPI) network among DEGs modulated by IL4-EVs or MSC-EVs compared to the CTRL group and its functional organization (Fig. 4A). The Molecular Complex Detection (MCODE) algorithm was applied to identify densely connected components. Each MCODE cluster was then subjected to independent enrichment analysis to determine the top-scoring terms that best describe the associated biological functions. This analysis identified eight significant MCODE clusters, each comprising gene subsets whose protein products physically interact with at least one other protein in the list (Fig. 4B). Interestingly, the MCODE clusters were found to be related to respiratory electron transport, mitochondrial translation, and ATPase complex, as well as proteasome and translation initiation factor activity (Fig. 4B). Hence, these results indicate that the pro-differentiating effects of microglial IL4 EVs and MSC-EVs on target OPCs involve a significant rewiring of mitochondrial functions.

### Upstream Regulators Driving the Effects of Pro-Regenerative Microglia-Derived EVs on OPC Maturation

**Fig. 5:**
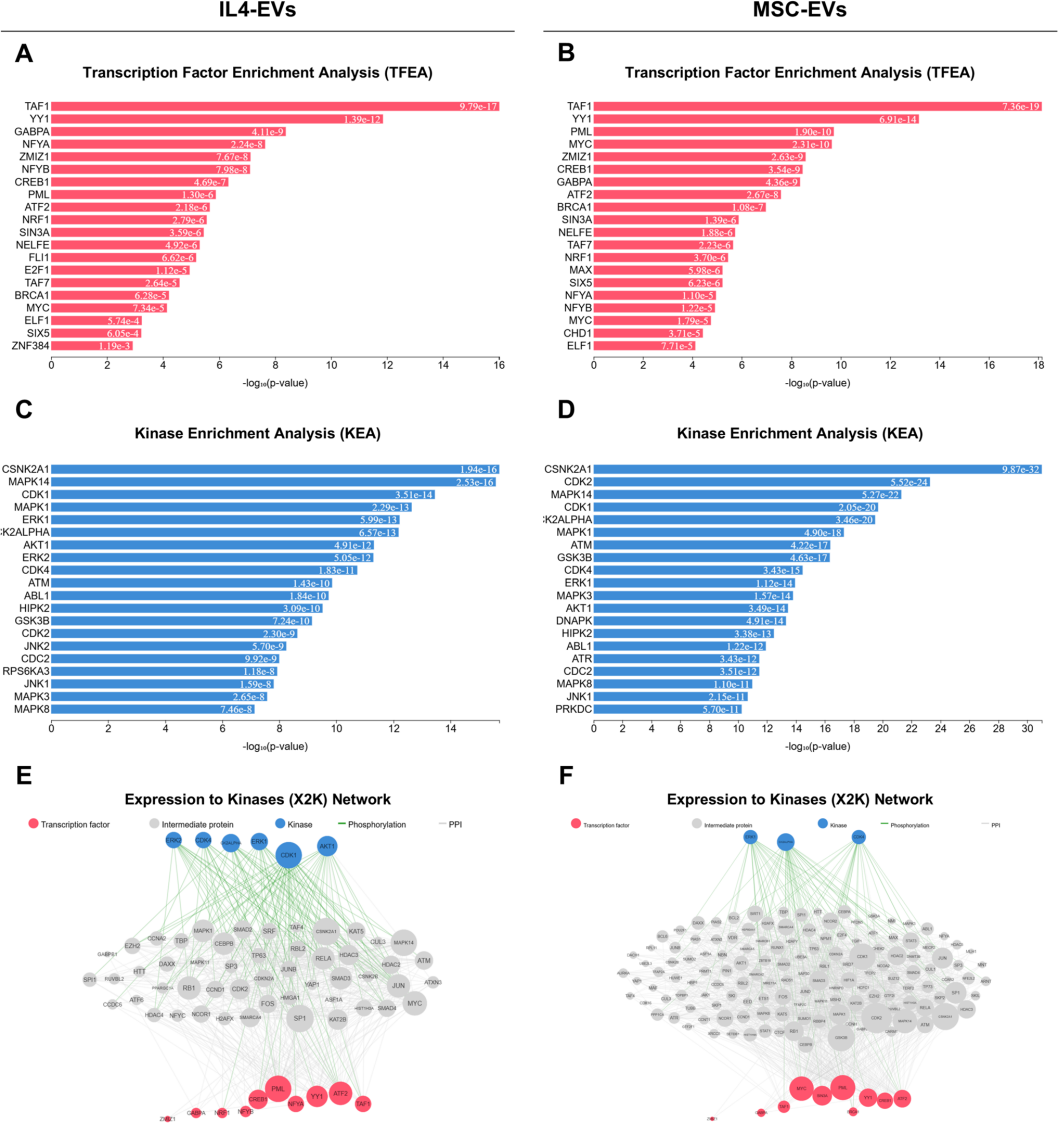
(**A-B**) Upstream transcription factors predicted by the X2K tool to regulate the transcriptional changes induced by IL4-EVs (A) or MSC-EVs (B) in recipient OPCs. (**C-D**) Upstream kinases predicted by the X2K tool to mediate the transcriptional changes induced by IL4-EVs (C) or MSC-EVs (D) in recipient OPCs. (**E–F**) X2K upstream regulatory network visualization for IL4-EVs (E) and MSC-EVs (F), depicting transcription factors (red nodes), intermediate protein/transcription factor interactors (white nodes), and kinases (blue nodes), with connecting lines indicating protein–protein interactions (PPIs) between factors and their interactors (white lines) and phosphorylation of kinases (green lines)

Finally, the X2K web tool (Clarke et al. 2018) has been exploited to infer the upstream regulatory networks responsible for the transcriptional changes induced by IL4-EVs and MSC-EVs in recipient OPCs. This approach revealed a large overlap between the two datasets in the top hit transcription factors (Fig. 5A, B), including TAF1 (targeting 158 and 207 DEGs, respectively) and YY1 (targeting 128 and 167 DEGs, respectively). Additionally, this analysis showed common top hit upstream kinases across the two datasets (Fig. 5C, D), including CSNK2A1 (targeting 36 and 84 DEGs, respectively), MAPK14 (targeting 30 and 58 DEGs, respectively), and CDK1 (targeting 35 and 73 DEGs, respectively). Visualization of full upstream regulatory networks, formed by predicted kinase-intermediate protein-transcription factor axes, allowed the identification of common hub transcription factors, such as TAF1, YY1, PML, GABPA, CREB1, ATF2, and ZMLZ1, and shared hub kinases, including CDK4. ERK1, and CK2ALPHA, across the two datasets (Fig. 5E, F). Altogether, these results indicate that IL4-EVs and MSC-EVs converge on common regulatory networks in OPCs, highlighting key transcription factors and kinases that could play pivotal roles in driving oligodendrocyte differentiation and may serve as potential therapeutic targets.

## Discussion

In this study, we provide a comprehensive comparative analysis of the transcriptional responses of OPCs to distinct microglial EV subtypes. We demonstrate that EVs derived from pro-regenerative microglia, specifically induced by stimulation with IL4 or co-culture with MSCs, promote the maturation of recipient OPCs by transcriptional reprogramming of mitochondrial and bioenergetic pathways. In contrast, EVs from pro-inflammatory microglia (i-EVs) induced minimal transcriptional changes compared to CTRL. Importantly, IL4-EVs and MSC-EVs elicited profound shifts in gene expression, converging on processes related to oxidative phosphorylation, mitochondrial organization, and translational machinery, revealing shared pathways triggered by these EV subtypes. Computational inference of upstream regulatory networks further revealed overlapping transcription factors and kinases, such as TAF1, YY1, CSNK2A1, and MAPK14, as candidate regulators of OPC differentiation modulated by microglial EVs. Together, these findings advance our understanding of how microglial EV heterogeneity shapes OPC transcriptional programs and identify mitochondrial rewiring as a central mechanism underlying microglial EV-driven OPC maturation, offering new targets for remyelination therapies.

The essential role of mitochondria in oligodendrocyte differentiation has gained increasing recognition. OPC maturation demands high energy expenditure for lipid biosynthesis and membrane assembly (Rosko et al. 2019; Marangon et al. 2020; Tepavčević 2021). Accordingly, prior work has linked mitochondrial dysfunction to remyelination failure (Madsen et al. 2017; de Barcelos et al. 2019; Meyer and Rinholm 2021; López-Muguruza and Matute 2023). Here, we provide direct evidence that endogenous pro-regenerative signals delivered via microglial EVs regulate the expression of mitochondrial respiratory chain components and ATP-synthase subunits in OPCs. Notably, both IL4-EVs and MSC-EVs enhanced expression of genes encoding inner membrane and ribosomal proteins, suggesting coordinated augmentation of oxidative metabolism and mitochondrial translation. This dual modulation may accelerate OPC bioenergetics, favoring the energy-intensive process of myelin sheath biogenesis.

Our results align with and extend previous studies on microglia–OPC crosstalk mediated by EVs. Microglial EV cargo, including bioactive proteins and lipids, has been implicated in OPC maturation and myelination (Lombardi et al. 2019, 2024; Raffaele et al. 2021b). Here, transcriptomic analysis reveals that these multifaceted signals converge on a common intracellular program centered on mitochondrial enhancement. This is in line with our previous data showing that membrane-bound TNF packaged in microglial EVs acts as a trophic cue by activating TNFR2 in recipient OPCs, which was shown to play a key role in regulating oligodendrocyte bioenergetics and subsequent maturation (Madsen et al. 2016; Raffaele et al. 2020, 2021b; Desu et al. 2021). In addition, EV-carried endocannabinoids, whose receptors are largely located in mitochondrial membranes, were shown to significantly promote OPC maturation, likely due to their impact on mitochondrial dynamics and energy homeostasis (Lipina et al. 2014; Molina-Holgado et al. 2023; Lombardi et al. 2024). The substantial overlap between IL4-EV and MSC-EV targets implies that, despite differences in EV composition, pro-regenerative microglial states deliver a conserved pro-differentiation message to OPCs. In contrast, i-EVs, enriched in inflammatory mediators, failed to drive the same metabolic shift, underscoring the importance of EV content in directing OPC fate.

Upstream network analysis pinpointed several transcription factors and kinases that may orchestrate mitochondrial gene programs in response to EV signals. The transcription factor YY1, previously implicated in regulating the transition of proliferating OPCs toward differentiation (He et al. 2007), has been highlighted by several studies as a key actor in mitochondrial bioenergetics required for the regeneration of skeletal muscles (Cunningham et al. 2007; Blättler et al. 2012; Chen et al. 2019), although its specific impact on oligodendrocyte energy metabolism has never been investigated. On the other end, inhibition of TAF1, a transcriptional co-activator associated with neurodevelopmental phenotypes and intellectual disability whose function in oligodendrocytes is still unknown (Crombie et al. 2024), has been previously shown to regulate the expression of genes involved in mitochondrial metabolism in cardiac cells (Leigh et al. 2023). Among kinases, MAPK14/p38 and CSNK2A1/CK2 are known regulators of stress responses and mitochondrial dynamics and their involvement in OPC differentiation has been already suggested by previous studies (Huillard et al. 2010; Chung et al. 2015; Haines et al. 2015; Bastian et al. 2019). By identifying these nodes, our work provides testable hypotheses for the molecular intermediates linking EV uptake to OPC metabolic remodeling. Future functional assays, such as pharmacological inhibition or genetic manipulation of these factors in OPCs, will be instrumental in validating their roles and uncovering possible targets to modulate oligodendrocyte bioenergetics and remyelination.

Therapeutically, EV-based approaches offer distinct advantages over cell transplantation or systemic drug administration. EVs can cross biological barriers, exhibit low immunogenicity, and can be engineered for targeted delivery (Wiklander et al. 2019; Gabrielli et al. 2022b). Our findings suggest that loading EVs with cargo enhancing mitochondrial function could potentiate remyelination in vivo. Moreover, small molecules or peptides that mimic EV-induced activation of key transcription factors or kinases may serve as mitochondria-targeted therapeutics. Importantly, the robust convergence of IL4-EVs and MSC-EVs on mitochondrial pathways indicates that diverse regenerative stimuli could be harnessed to produce EVs with similar efficacies, broadening their translational potential.

Several limitations should be acknowledged. First, our transcriptomic profiling was performed at an early differentiation time point (24 h) in vitro to catch the first changes induced by exposure to microglial EVs, which may not fully capture the temporal dynamics of gene expression during OPC maturation and myelination. Longitudinal studies integrating proteomic and metabolomic analyses would deepen insight into the functional consequences of early transcriptional rewiring. Second, although our bioinformatic analyses suggested candidate pathways, transcription factors, and kinases as potential regulators of OPC maturation mediated by microglial EVs, here we did not perform direct validation of these targets at the protein level, which limits the ability to confirm their functional relevance in vitro or in vivo. Third, in the present study we did not directly quantify EV number or protein content, so subtle differences in EV yield between conditions cannot be fully excluded; however, the treatment design based on donor-to-recipient cell ratios was informed by our previous work showing comparable EV secretion across conditions (Lombardi et al. 2019; Raffaele et al. 2021b). Last, the heterogeneity of EV subpopulations (based on their different size and biogenesis) and their individual contributions remain to be elucidated. Future efforts combining high-resolution EV isolation with single-vesicle analysis could refine our understanding of the active EV species.

In conclusion, this work uncovers mitochondrial rewiring as a fundamental mechanism by which pro-regenerative microglial EVs promote OPC differentiation. By defining the transcriptomic landscape and upstream regulatory networks induced in target OPCs, we lay the groundwork for developing EV-based, mitochondria-targeted interventions to enhance remyelination in diverse CNS disorders. Further exploration of microglial EV-OPC signaling axes promises to yield novel strategies for preserving white matter integrity and fighting neurological disabilities.

## Supplementary Information

Below is the link to the electronic supplementary material.

Supplementary file1 (XLSX 319 KB)

Supplementary file2 (XLSX 243 KB)

## Data Availability

Microarray raw data are available in the GEO database (accession number: GSE304130). Further information and data that support the findings of this study are available from the corresponding authors upon reasonable request.

## References

[CR1] Ashburner M, Ball CA, Blake JA et al (2000) Gene ontology: tool for the unification of biology. Nat Genet. 10.1038/75556 10802651 10.1038/75556 PMC3037419

[CR2] Back SA (2017) White matter injury in the preterm infant: pathology and mechanisms. Acta Neuropathol 134:331 28534077 10.1007/s00401-017-1718-6 PMC5973818

[CR3] Bader GD, Hogue CWV (2003) An automated method for finding molecular complexes in large protein interaction networks. BMC Bioinformatics. 10.1186/1471-2105-4-2 12525261 10.1186/1471-2105-4-2 PMC149346

[CR4] Bastian C, Quinn J, Tripathi A et al (2019) CK2 inhibition confers functional protection to young and aging axons against ischemia by differentially regulating the CDK5 and AKT signaling pathways. Neurobiol Dis 126:47–61. 10.1016/j.nbd.2018.05.011 29944965 10.1016/j.nbd.2018.05.011 PMC9084539

[CR5] Bianco F, Pravettoni E, Colombo A et al (2005) Astrocyte-derived ATP induces vesicle shedding and IL-1β release from microglia. J Immunol 174:7268–7277. 10.4049/jimmunol.174.11.7268 15905573 10.4049/jimmunol.174.11.7268

[CR6] Blättler SM, Verdeguer F, Liesa M et al (2012) Defective mitochondrial morphology and bioenergetic function in mice lacking the transcription factor Yin Yang 1 in skeletal muscle. Mol Cell Biol. 10.1128/mcb.00337-12 22711985 10.1128/MCB.00337-12 PMC3434543

[CR7] Bonfanti E, Bonifacino T, Raffaele S et al (2020) Abnormal upregulation of GPR17 receptor contributes to oligodendrocyte dysfunction in SOD1 G93A mice. Int J Mol Sci 21:2395. 10.3390/IJMS21072395 32244295 10.3390/ijms21072395 PMC7177925

[CR8] Chen F, Zhou J, Li Y et al (2019) YY 1 regulates skeletal muscle regeneration through controlling metabolic reprogramming of satellite cells. EMBO J. 10.15252/embj.201899727 30979776 10.15252/embj.201899727 PMC6518041

[CR9] Cheng YJ, Wang F, Feng J et al (2024) Prolonged myelin deficits contribute to neuron loss and functional impairments after ischaemic stroke. Brain 147:1294–1311. 10.1093/brain/awae029 38289861 10.1093/brain/awae029

[CR10] Chung S-H, Biswas S, Selvaraj V et al (2015) The p38α mitogen-activated protein kinase is a key regulator of myelination and remyelination in the CNS. Cell Death Dis 6:e1748–e1748. 10.1038/cddis.2015.119 25950478 10.1038/cddis.2015.119 PMC4669698

[CR11] Clarke DJB, Kuleshov MV, Schilder BM et al (2018) EXpression2Kinases (X2K) Web: linking expression signatures to upstream cell signaling networks. Nucleic Acids Res. 10.1093/nar/gky458 29800326 10.1093/nar/gky458 PMC6030863

[CR12] Crombie EM, Cleverley K, Timmers HTM, Fisher EMC (2024) The roles of TAF1 in neuroscience and beyond. R Soc Open Sci. 10.1098/rsos.240790 39323550 10.1098/rsos.240790 PMC11423858

[CR13] Cunningham JT, Rodgers JT, Arlow DH et al (2007) mTOR controls mitochondrial oxidative function through a YY1-PGC-1α transcriptional complex. Nature. 10.1038/nature06322 18046414 10.1038/nature06322

[CR14] de Barcelos IP, Troxell RM, Graves JS (2019) Mitochondrial dysfunction and multiple sclerosis. Biology 8:37. 10.3390/biology8020037 31083577 10.3390/biology8020037 PMC6627385

[CR15] Desu HL, Illiano P, Choi JS et al (2021) TNFR2 signaling regulates the immunomodulatory function of oligodendrocyte precursor cells. Cells 10:1785. 10.3390/cells10071785 34359956 10.3390/cells10071785 PMC8306473

[CR16] Festa LK, Grinspan JB, Jordan-Sciutto KL (2024) White matter injury across neurodegenerative disease. Trends Neurosci 47:47–57. 10.1016/j.tins.2023.11.003 38052682 10.1016/j.tins.2023.11.003 PMC10842057

[CR17] Franklin RJM, Ffrench-Constant C (2017) Regenerating CNS myelin - from mechanisms to experimental medicines. Nat Rev Neurosci 18:753–769 29142295 10.1038/nrn.2017.136

[CR18] Gabrielli M, Battista N, Riganti L et al (2015) Active endocannabinoids are secreted on extracellular membrane vesicles. EMBO Rep 16:213–220. 10.15252/embr.201439668 25568329 10.15252/embr.201439668 PMC4328748

[CR19] Gabrielli M, Prada I, Joshi P et al (2022a) Microglial large extracellular vesicles propagate early synaptic dysfunction in Alzheimer’s disease. Brain. 10.1093/BRAIN/AWAC083 35254410 10.1093/brain/awac083 PMC9420022

[CR20] Gabrielli M, Raffaele S, Fumagalli M, Verderio C (2022b) The multiple faces of extracellular vesicles released by microglia: where are we 10 years after? Front Cell Neurosci. 10.3389/FNCEL.2022.984690 36176630 10.3389/fncel.2022.984690 PMC9514840

[CR21] Gualerzi A, Lombardi M, Verderio C (2021) Microglia-oligodendrocyte intercellular communication: role of extracellular vesicle lipids in functional signalling. Neural Regen Res 16:1194. 10.4103/1673-5374.300430 33269772 10.4103/1673-5374.300430 PMC8224135

[CR22] Haines JD, Fulton DL, Richard S, Almazan G (2015) P38 mitogen-activated protein kinase pathway regulates genes during proliferation and differentiation in oligodendrocytes. PLoS ONE 10:e0145843. 10.1371/journal.pone.0145843 26714323 10.1371/journal.pone.0145843 PMC4699908

[CR23] He Y, Dupree J, Wang J et al (2007) The transcription factor Yin Yang 1 is essential for oligodendrocyte progenitor differentiation. Neuron. 10.1016/j.neuron.2007.06.029 17640524 10.1016/j.neuron.2007.06.029 PMC2034312

[CR24] Huillard E, Ziercher L, Blond O et al (2010) Disruption of *CK2* β in embryonic neural stem cells compromises proliferation and oligodendrogenesis in the mouse telencephalon. Mol Cell Biol 30:2737–2749. 10.1128/MCB.01566-09 20368359 10.1128/MCB.01566-09 PMC2876519

[CR25] Irizarry RA, Bolstad BM, Collin F et al (2003) Summaries of Affymetrix GeneChip probe level data. Nucleic Acids Res 31:e15. 10.1093/nar/gng015 12582260 10.1093/nar/gng015 PMC150247

[CR26] Kanehisa M, Furumichi M, Sato Y et al (2023) KEGG for taxonomy-based analysis of pathways and genomes. Nucleic Acids Res. 10.1093/nar/gkac963 36300620 10.1093/nar/gkac963 PMC9825424

[CR27] Kedia S, Simons M (2025) Oligodendrocytes in Alzheimer’s disease pathophysiology. Nat Neurosci 28:446–456. 10.1038/s41593-025-01873-x 39881195 10.1038/s41593-025-01873-x

[CR28] Keenan AB, Torre D, Lachmann A et al (2019) ChEA3: transcription factor enrichment analysis by orthogonal omics integration. Nucleic Acids Res. 10.1093/nar/gkz446 31114921 10.1093/nar/gkz446 PMC6602523

[CR29] Kent SA, Miron VE (2023) Microglia regulation of central nervous system myelin health and regeneration. Nat Rev Immunol 2023:1–15. 10.1038/s41577-023-00907-4 10.1038/s41577-023-00907-4 37452201

[CR30] Kuleshov MV, Xie Z, London ABK et al (2021) Kea3: improved kinase enrichment analysis via data integration. Nucleic Acids Res. 10.1093/nar/gkab359 34019655 10.1093/nar/gkab359 PMC8265130

[CR31] Leigh RS, Välimäki MJ, Kaynak BL, Ruskoaho HJ (2023) TAF1 bromodomain inhibition as a candidate epigenetic driver of congenital heart disease. Biochimica Et Biophysica Acta (BBA) - Mol Basis Dis 1869:166689. 10.1016/j.bbadis.2023.166689 10.1016/j.bbadis.2023.166689 36958711

[CR32] Li Y, Liu Z, Song Y et al (2022) M2 microglia-derived extracellular vesicles promote white matter repair and functional recovery via miR-23a-5p after cerebral ischemia in mice. Theranostics 12:3553–3573. 10.7150/THNO.68895 35547763 10.7150/thno.68895 PMC9065182

[CR33] Lipina C, Irving AJ, Hundal HS (2014) Mitochondria: a possible nexus for the regulation of energy homeostasis by the endocannabinoid system? Am J Physiol Endocrinol Metab. 10.1152/ajpendo.00100.2014 24801388 10.1152/ajpendo.00100.2014

[CR34] Lloyd AF, Miron VE (2019) The pro-remyelination properties of microglia in the central nervous system. Nat Rev Neurol. 10.1038/s41582-019-0184-2 31256193 10.1038/s41582-019-0184-2

[CR35] Lombardi M, Parolisi R, Scaroni F et al (2019) Detrimental and protective action of microglial extracellular vesicles on myelin lesions: astrocyte involvement in remyelination failure. Acta Neuropathol 138:987–1012. 10.1007/s00401-019-02049-1 31363836 10.1007/s00401-019-02049-1 PMC6851224

[CR36] Lombardi M, Scaroni F, Gabrielli M et al (2024) Extracellular vesicles released by microglia and macrophages carry endocannabinoids which foster oligodendrocyte differentiation. Front Immunol. 10.3389/fimmu.2024.1331210 38464529 10.3389/fimmu.2024.1331210 PMC10921360

[CR37] López-Muguruza E, Matute C (2023) Alterations of oligodendrocyte and myelin energy metabolism in multiple sclerosis. Int J Mol Sci. 10.3390/ijms241612912 37629092 10.3390/ijms241612912 PMC10454078

[CR38] Madsen PM, Motti D, Karmally S et al (2016) Oligodendroglial TNFR2 mediates membrane TNF-dependent repair in experimental autoimmune encephalomyelitis by promoting oligodendrocyte differentiation and remyelination. J Neurosci 36:5128–5143. 10.1523/JNEUROSCI.0211-16.2016 27147664 10.1523/JNEUROSCI.0211-16.2016 PMC4854972

[CR39] Madsen PM, Pinto M, Patel S et al (2017) Mitochondrial DNA double-strand breaks in oligodendrocytes cause demyelination, axonal injury, and CNS inflammation. J Neurosci 37:10185–10199. 10.1523/JNEUROSCI.1378-17.2017 28931570 10.1523/JNEUROSCI.1378-17.2017 PMC5647772

[CR40] Marangon D, Boccazzi M, Lecca D, Fumagalli M (2020) Regulation of oligodendrocyte functions: targeting lipid metabolism and extracellular matrix for myelin repair. J Clin Med 9:470. 10.3390/jcm9020470 32046349 10.3390/jcm9020470 PMC7073561

[CR41] Marangon D, Audano M, Pedretti S et al (2022) Rewiring of glucose and lipid metabolism induced by G protein-coupled receptor 17 silencing enables the transition of oligodendrocyte progenitors to myelinating cells. Cells 11:2369. 10.3390/cells11152369 35954217 10.3390/cells11152369 PMC9368002

[CR42] Meyer N, Rinholm JE (2021) Mitochondria in myelinating oligodendrocytes: slow and out of breath? Metabolites. 10.3390/metabo11060359 34198810 10.3390/metabo11060359 PMC8226700

[CR43] Milacic M, Beavers D, Conley P et al (2024) The Reactome pathway knowledgebase 2024. Nucleic Acids Res. 10.1093/nar/gkad1025 37941124 10.1093/nar/gkad1025 PMC10767911

[CR44] Molina-Gonzalez I, Miron VE, Antel JP (2022) Chronic oligodendrocyte injury in central nervous system pathologies. Commun Biol 5:1274. 10.1038/s42003-022-04248-1 36402839 10.1038/s42003-022-04248-1 PMC9675815

[CR45] Molina-Holgado E, Esteban PF, Arevalo-Martin Á et al (2023) Endocannabinoid signaling in oligodendroglia. Glia. 10.1002/glia.24180 35411970 10.1002/glia.24180

[CR46] Oughtred R, Stark C, Breitkreutz BJ et al (2019) The BioGRID interaction database: 2019 update. Nucleic Acids Res. 10.1093/nar/gky1079 30476227 10.1093/nar/gky1079 PMC6324058

[CR47] Prada I, Gabrielli M, Turola E et al (2018) Glia-to-neuron transfer of miRNAs via extracellular vesicles: a new mechanism underlying inflammation-induced synaptic alterations. Acta Neuropathol 135:529–550. 10.1007/s00401-017-1803-x 29302779 10.1007/s00401-017-1803-x PMC5978931

[CR48] Raffaele S, Fumagalli M (2022) Dynamics of microglia activation in the ischemic brain : implications for myelin repair and functional recovery. Front Cell Neurosci 16:1–10. 10.3389/fncel.2022.950819 10.3389/fncel.2022.950819 PMC9309466 35899017

[CR49] Raffaele S, Lombardi M, Verderio C, Fumagalli M (2020) TNF production and release from microglia via extracellular vesicles: impact on brain functions. Cells 9:2145 32977412 10.3390/cells9102145 PMC7598215

[CR50] Raffaele S, Boccazzi M, Fumagalli M (2021a) Oligodendrocyte dysfunction in amyotrophic lateral sclerosis: mechanisms and therapeutic perspectives. Cells 10:565. 10.3390/cells10030565 33807572 10.3390/cells10030565 PMC8000560

[CR51] Raffaele S, Gelosa P, Bonfanti E et al (2021b) Microglial vesicles improve post-stroke recovery by preventing immune cell senescence and favoring oligodendrogenesis. Mol Ther 29:1439–1458. 10.1016/j.ymthe.2020.12.009 33309882 10.1016/j.ymthe.2020.12.009 PMC8058432

[CR52] Rosko L, Smith VN, Yamazaki R, Huang JK (2019) Oligodendrocyte bioenergetics in health and disease. Neuroscientist 25:334–343 30122106 10.1177/1073858418793077 PMC6745601

[CR53] Szklarczyk D, Kirsch R, Koutrouli M et al (2023) The STRING database in 2023: protein-protein association networks and functional enrichment analyses for any sequenced genome of interest. Nucleic Acids Res. 10.1093/nar/gkac1000 36370105 10.1093/nar/gkac1000 PMC9825434

[CR54] Tepavčević V (2021) Oligodendroglial energy metabolism and (Re)myelination. Life. 10.3390/life11030238 33805670 10.3390/life11030238 PMC7998845

[CR55] Vanherle S, Guns J, Loix M et al (2023) Extracellular vesicle-associated cholesterol supports the regenerative functions of macrophages in the brain. J Extracell Vesicles. 10.1002/jev2.12394 38124258 10.1002/jev2.12394 PMC10733568

[CR56] Welsh JA, Goberdhan DCI, O’Driscoll L et al (2024) Minimal information for studies of extracellular vesicles (MISEV2023): from basic to advanced approaches. J Extracell Vesicles. 10.1002/jev2.12404 38326288 10.1002/jev2.12404 PMC10850029

[CR57] Wiklander OPB, Brennan M, Lötvall J et al (2019) Advances in therapeutic applications of extracellular vesicles. Sci Transl Med 11:1–16. 10.1126/scitranslmed.aav8521 10.1126/scitranslmed.aav8521 PMC7104415 31092696

[CR58] Zappia E, Casazza S, Pedemonte E et al (2005) Mesenchymal stem cells ameliorate experimental autoimmune encephalomyelitis inducing t-cell anergy. Blood 106:1755–1761. 10.1182/blood-2005-04-1496 15905186 10.1182/blood-2005-04-1496

[CR59] Zhou Y, Zhou B, Pache L et al (2019) Metascape provides a biologist-oriented resource for the analysis of systems-level datasets. Nat Commun. 10.1038/s41467-019-09234-6 30944313 10.1038/s41467-019-09234-6 PMC6447622

